# Comparison of Nine Statistical Model Based Warfarin Pharmacogenetic Dosing Algorithms Using the Racially Diverse International Warfarin Pharmacogenetic Consortium Cohort Database

**DOI:** 10.1371/journal.pone.0135784

**Published:** 2015-08-25

**Authors:** Rong Liu, Xi Li, Wei Zhang, Hong-Hao Zhou

**Affiliations:** 1 Department of Clinical Pharmacology, Xiangya Hospital, Central South University, Changsha, P. R. China; 2 Institute of Clinical Pharmacology, Central South University, Hunan Key Laboratory of Pharmacogenetics, Changsha, P. R. China; National Institute of Genomic Medicine, MEXICO

## Abstract

**Objective:**

Multiple linear regression (MLR) and machine learning techniques in pharmacogenetic algorithm-based warfarin dosing have been reported. However, performances of these algorithms in racially diverse group have never been objectively evaluated and compared. In this literature-based study, we compared the performances of eight machine learning techniques with those of MLR in a large, racially-diverse cohort.

**Methods:**

MLR, artificial neural network (ANN), regression tree (RT), multivariate adaptive regression splines (MARS), boosted regression tree (BRT), support vector regression (SVR), random forest regression (RFR), lasso regression (LAR) and Bayesian additive regression trees (BART) were applied in warfarin dose algorithms in a cohort from the International Warfarin Pharmacogenetics Consortium database. Covariates obtained by stepwise regression from 80% of randomly selected patients were used to develop algorithms. To compare the performances of these algorithms, the mean percentage of patients whose predicted dose fell within 20% of the actual dose (mean percentage within 20%) and the mean absolute error (MAE) were calculated in the remaining 20% of patients. The performances of these techniques in different races, as well as the dose ranges of therapeutic warfarin were compared. Robust results were obtained after 100 rounds of resampling.

**Results:**

BART, MARS and SVR were statistically indistinguishable and significantly out performed all the other approaches in the whole cohort (MAE: 8.84–8.96 mg/week, mean percentage within 20%: 45.88%–46.35%). In the White population, MARS and BART showed higher mean percentage within 20% and lower mean MAE than those of MLR (all *p* values < 0.05). In the Asian population, SVR, BART, MARS and LAR performed the same as MLR. MLR and LAR optimally performed among the Black population. When patients were grouped in terms of warfarin dose range, all machine learning techniques except ANN and LAR showed significantly higher mean percentage within 20%, and lower MAE (all *p* values < 0.05) than MLR in the low- and high- dose ranges.

**Conclusion:**

Overall, machine learning-based techniques, BART, MARS and SVR performed superior than MLR in warfarin pharmacogenetic dosing. Differences of algorithms’ performances exist among the races. Moreover, machine learning-based algorithms tended to perform better in the low- and high- dose ranges than MLR.

## Introduction

Warfarin is a widely used oral anticoagulant agent with a narrow therapeutic window and extremely wide inter-individual variability in dose requirement [1]. The consequences of inadequate dosing include over-anticoagulation or hemorrhage, as well as recurrence of the thrombotic event for which the drug was indicated and developed. Much effort has been devoted to improve warfarin dose recommendations and reduce the unpredictability of warfarin response [2], such attempts include adjustment of warfarin dose based on the measurements of the international normalized ratio and development of new ways to determine the appropriate warfarin dose. Non-genetic and genetic factors significantly contribute to inter-individual variability in warfarin dose requirement. Non-genetic factors, such as age, height, weight, race and the use of drugs interacting with warfarin, have been reported to affect the variability of responses to warfarin [3–6]. Furthermore, genetic factors are considered determinants of warfarin dose requirement. Particularly, polymorphisms in cytochrome P450 2C9 (*CYP2C9*) and vitamin K epoxide reductase complex 1 (*VKORC1*) genes have generally contributed to 6–18% and 15–30% of warfarin dose variability, respectively [7–12]. *CYP2C9* polymorphisms alter the pharmacokinetics of warfarin, whereas *VKORC1* polymorphisms affect its pharmacodynamics.

Previous studies have developed predictive pharmacogenetic dosing algorithms for warfarin, and the results showed that the algorithms predicted 37–55% of the patient’s warfarin stable dose (WSD) [3, 5, 6, 9, 12–20]. Most of the dosing algorithms mentioned above are based on multiple linear regression (MLR) methods, which are commonly employed to obtain dosing data [3]. Moreover, these pharmacogenetic algorithms are derived from different racial groups, with therapeutic WSD acting as dependent variable, and several genetic and non-genetic factors as independent variables. However, MLR demonstrates some well-known limitations that may affect the prediction accuracy. More importantly, the relationship between the dependent and independent variables is complex and non-linear. For example, previous investigations proved that the interaction between *CYP2C9* and *VKORC1* genotypes is related to the outcomes of anticoagulant drugs, such as in the maintenance dose for phenprocoumon [21] and warfarin [22], hence, MLR may not be most feasible method to accurately predict the outcomes of these drugs [23].

Other machine learning techniques have been tested to predict the optimal warfarin maintenance dose because of their advantages, including lack of parametric assumptions, high power and flexibility. Three machine learning approaches, namely, random forest regression (RFR), boosted regression tree (BRT) and support vector regression (SVR), were employed to predict warfarin maintenance dose in a cohort of African Americans; many genotypic variables were incorporated and the *R* ^*2*^ between the predicted and actual square root of warfarin dose in this model showed an average 66.4% for RFR, 57.8% for SVR, and 56.9% for BRT, compared with 27% reported by the International Warfarin Pharmacogenetics Consortium (IWPC) [24] for African Americans [25]. Artificial neural network (ANN) has also been applied and appears to be a promising tool in warfarin maintenance dose prediction with an average absolute error of 5.7 mg/week [26].

To date, identifying which algorithm performs better (either MLR or machine learning techniques based algorithms) is difficult. On the one hand, several predictors are currently used in published warfarin pharmacogenetic algorithms. On the other hand, these algorithms are derived from studies involving cohorts with different racial backgrounds. Little information is available on the comparative performance of machine learning and MLR-based warfarin pharmacogenetic algorithms, and only few of these algorithms have been compared for predictive accuracy in racially homogeneous and small populations [25, 27, 28]. The present study aimed to perform a systematic review of the current literature to compare the performance of warfarin machine learning pharmacogenetic techniques with that of MLR and evaluate these algorithms in terms of race and therapeutic warfarin dose range.

## Materials and Methods

### Literature Search and Algorithm Selection

To identify publications on machine learning warfarin pharmacogenetic dosing algorithm, we conducted a web-based literature search in PubMed using various combinations of the following keywords: ‘warfarin’, ‘machine learning’, ‘pharmacogenetics’, ‘*CYP2C9*’, ‘*VKORC1*’ and ‘data mining’. Our literature search was limited to journal articles published before May 31, 2014. From among the publications on WSD prediction, Six reported machine learning techniques were selected, namely ANN [26], regression tree (RT) [29], multivariate adaptive regression splines (MARS) [3], BRT, SVR and RFR [25]. In addition to the six machine learning based techniques mentioned above, two classical machine learning techniques, namely, lasso regression (LAR) and Bayesian additive regression trees (BART) and the most widely used MLR were included in our study for comparison.

### The International Warfarin Pharmacogenetic Consortium Cohort

IWPC open access data downloaded from the PharmGKB website (http://www.pharmgkb.org/downloads/) were used to develop and compare the algorithms, data from the website included 6256 patients treated with warfarin. Patients were recruited by 22 research groups from nine countries in four continents. Genetic and non-genetic information, including patient clinical information, concomitant medications, therapeutic doses, and their *CYP2C9* and *VKORC1* rs9923231 (-1639G/A) genotypes were recorded and supplied [3].

Prior to analysis, we excluded the following patients: 1) those lacking of non-genetic information (height, weight and age), which is necessary to calculate warfarin stable dose; 2) patients lacking genotype information about their *CYP2C9* or *VKORC1* rs9923231 genotypes; and 3) those who have not yet achieved warfarin stable dosage. Finally, a total of 4798 patients were selected for subsequent analysis. They were divided into four cohorts, White, Asian, Black, and missing or mixed race, with a population of 2718, 1156, 665 and 259, respectively. Considering the small sample size of missing or mixed race, comparative analysis by race was conducted among the three other cohorts only.

### Comparison of Performances of the Algorithms

Performances of the algorithms were compared using two evolution indexes, namely, mean absolute error and the percentage of patients whose predicted warfarin dose was within 20% of the actual dose in the validating cohort. The mean absolute error (MAE) is the average of the absolute value of predicted dose minus the actual dose. We selected the percentage of patients within 20% of the actual dose (percentage within 20%) because a change in warfarin dose greater than 20% may be considered clinically significant, and this definition has been widely accepted and applied [4]. The MAE and percentage within 20% of the algorithms were also compared in terms of race and warfarin dose range. Warfarin dose range was divided into three categories based on the 25% and 75% quantiles of WSD by races: low dose (≤14 mg/week), intermediate dose (14–26.25 mg/week), and high dose (>26.25 mg/week) in the Asian population; low dose (≤22 mg/week), intermediate dose (22–42.49 mg/week), and high dose (>42.49 mg/week) in the White cohort; low dose (≤30 mg/week), intermediate dose (30–52.5 mg/week), and high dose (>52.5 mg/week) in the Black cohort; and low dose (≤22.5 mg/week), intermediate dose (22.5–40 mg/week), and high dose (>40 mg/week) in the missing or mixed race.

### Statistical Analyses

Descriptive statistics was used to determine frequency distributions, percentage distributions, means and standard deviations. Chi-square test was used to assess deviations of allele frequencies from Hardy-Weinberg equilibrium.

All the algorithms were implemented using R statistical software. To develop warfarin dose algorithms in the training cohort, stepwise regression was used to select the covariates related to WSD, as the dependent variable in the prediction models. In the entire cohort, covariates including race, *VKORC1* and *CYP2C9* genotypes, age in years, weight in kg, height in cm, smoking history, amiodarone use, and use of enzyme inducer were used as independent variables; in the Asian cohort, the covariates included *VKORC1* and *CYP2C9* genotypes, age in years, weight in kg, amiodarone use, and smoking history; and in the White and Black cohorts, the predictors were *VKORC1* and *CYP2C9* genotypes, age in years, weight in kg, amiodarone use, smoking history, and enzyme inducer use. Given that the WSD data were not normally distributed, the square root of transformed weekly WSD was set as dependent variable in all the prediction models. We used the rpart package for RT, RSNNS package for ANN, gbm package for BRT, randomForest package for RFR, earth package for MARS, e1071 package for SVR, bartMachine package for BART and glmnet package for LAR. Default parameters were used.

To obtain robust results, resampling was performed. In the entire cohort, we randomly selected 80% (3838 patients) among the eligible patients, as the “derivation cohort” to develop all dose-prediction algorithms. The remaining 20% of the patients (960 patients) constituted the “validation cohort,” which was used to test the final selected algorithms. The MAE and mean percentage within 20% in the whole population, as well as in terms of warfarin dose range were obtained after 100 rounds of resampling. Furthermore, 95% confidence interval (CI) of MAE was calculated. Similar resampling processes were conducted with regard to race. To test the differences of the mean percentage within 20% among these algorithms, two independent sample *t*-tests were performed.

To determine a correlation between the average MAE and mean percentage within 20%, Spearman’s correlation test was performed. All the above analyses were conducted with R (Version 3.1.0).

## Results

### Basic Characteristics of the Study Cohorts

A total of 4798 patients were included in our study. Among the 6256 patients in the IWPC database, 1458 patients were excluded because of missing genetic information (*CYP2C9* or *VKORC*1 genotype) or non-genetic information (e.g. height, weight, or age), which are both necessary to calculate the warfarin stable dose.

The characteristics of the 4798 patients are listed in Table 1. Among the patients, 83.64% were aged 50 years or older. The mean and standard deviation of WSD were 32.33 and 17.42 mg/week, respectively. Amiodarone was administered in 220 patients, whereas enzyme inducers, such as carbamazepine, phenytoin, rifampin or rifampicin were administered in 51 patients. About 73.97% of the total population was homozygous for the CYP2C9*1 allele, whereas 4.17% comprised non-carriers of this wild-type allele. The frequencies of *VKORC1-1639* genotypes A/A, A/G and G/G were 26.82%, 30.83% and 30.14%, respectively. The allelic frequencies of both genotypes when participants were grouped in terms of race were shown in Hardy–Weinberg equilibrium.

**Table 1:** Basic characteristics of International Warfarin Pharmacogenetics Consortium patients included in this study.

Variable	IWPC data
	(n = 4798)
Warfarin dose—(mg/week);mean(SD)	32.33 (17.42)
Height–(cm); mean(SD)	168.42 (10.85)
Weight–(kg); mean(SD)	79.54 (22.53)
Population by race—(%)	
Asian	1156 (24.09)
White	2718 (56.65)
Black	665 (13.86)
Missing or mixed race	259 (5.40)
Genotype (%)	
VKORC1 rs9923231	
A/A	1287 (26.82)
A/G	1479 (30.83)
G/G	1446 (30.14)
Unknown	586 (12.21)
CYP2C9	
*1/*1	3549 (73.97)
*1/*2	646 (13.46)
*1/*3	394 (8.21)
*1/*5	6 (0.13)
*1/*6	3 (0.06)
*2/*2	50 (1.04)
*2/*3	57 (1.19)
*3/*3	12 (0.25)
Unknown	81 (1.69)
Population by age (%)	
<50	785 (16.36)
>50	4013 (83.64)
Number of females (%)	2027 (42.26)
Medications (%)	
Enzyme inducer*	51 (1.06)
Amiodarone	220 (4.59)
Smoker	440 (9.17)

*Enzyme inducer defined as carbamazepine, phenytoin, rifampin or rifampicin

### Overall Comparison of Predictive Algorithms

In the whole validation cohort, most of the algorithms yielded similar MAE (8. 84–9.82 mg/week) and mean percentage within 20% (41.27–46.35%) (Table 2). Some machine learning-based algorithms, including SVR, MARS and BART resulted in lower MAE and higher mean percentage within 20% (MAE ranged from 8.84 mg/week to 8.96 mg/week, mean percentage within 20% ranged from 45.88% to 46.35%) than those of all the other algorithms; *t*-test results showed that all *p* values were <0.05 (Table A in S1 File). Among the algorithms, MARS demonstrated optimal performance. By contrast, ANN performed the least feasible (average MAE was 9.82; mean percentage within 20% was 41.27%). The average MAE was inversely correlated with the percentage within 20% (Spearman’s correlation coefficient = −1, *p* value < 0.01).

**Table 2:** Mean absolute error and percentage within 20% of actual dose in validation cohorts. Data are expressed as mean (95% CI) or percentage.MAE: mean absolute error; MLR: multiple linear regression; SVR: support vector regression; ANN: artificial neural network; RT: regression tree; RFR: random forest regression; BRT: boosted regression tree; MARS: multivariate adaptive regression splines; LAR: lasso regression; BART: Bayesian additive regression trees. To build warfarin dose algorithms in the whole training cohort, the covariates were race, genotypes of *VKORC1* and *CYP2C9*, age in years, weight in kg, height in cm, smoking history, amiodarone use, and enzyme inducer use; in the Asian cohort, the covariates were genotypes of *VKORC1* and *CYP2C9*, age in years, weight in kg, amiodarone use, and smoking history; in the White and Black cohorts, the covariates were genotypes of *VKORC1* and *CYP2C9*, age in years, weight in kg, amiodarone use, smoking history, and enzyme inducer use.

Algorithms	All(n = 960)		Asian(n = 231)		White(n = 544)		Black(n = 133)	
	MAE	Within 20%	MAE	Within 20%	MAE	Within 20%	MAE	Within 20%
SVR	8.96 (8.33–9.60)	45.88	6.15 (5.39–6.90)	46.43	9.59 (8.65–10.53)	44.99	12.41 (10.44–14.39)	42.20
ANN	9.82 (9.16–10.49)	41.27	6.54 (5.77–7.31)	42.87	10.60 (9.62–11.57)	40.27	13.26 (11.20–15.32)	40.11
RT	9.57 (8.91–10.24)	43.04	6.55 (5.77–7.33)	42.37	10.59 (9.61–11.58)	40.69	13.84 (11.72–15.96)	37.46
MLR	9.28 (8.63–9.92)	43.97	6.17 (5.42–6.93)	46.16	10.00 (9.05–10.94)	42.77	12.17 (10.23–14.10)	43.24
RFR	9.05 (8.42–9.69)	45.34	6.26 (5.50–7.02)	45.16	9.70 (8.76–10.64)	44.00	12.69 (10.67–14.71)	41.56
BRT	8.95 (8.33–9.58)	45.57	6.26 (5.50–7.02)	45.29	9.58 (8.65–10.51)	44.60	12.55 (10.58–14.52)	42.16
MARS	8.84 (8.22–9.46)	46.35	6.10 (5.36–6.84)	46.56	9.36 (8.45–10.27)	46.22	12.33 (10.40–14.27)	42.15
LAR	9.28 (8.64–9.93)	43.95	6.18 (5.42–6.93)	46.10	10.00 (9.05–10.95)	42.67	12.17 (10.23–14.10)	43.30
BART	8.87 (8.25–9.50)	46.03	6.07 (5.32–6.81)	46.73	9.45 (8.53–10.37)	46.06	12.32 (10.40–14.24)	41.76

### Predictive Algorithm Comparison by Race

Comparison of the algorithms’ performances in terms of race is presented in Table 2. Overall, the difference in the mean percentage within 20% of the algorithms across the three cohorts was much smaller than that in the average MAE. All the algorithms yielded similar mean percentage within 20% across racial groups. Furthermore, either the machine learning or MLR based algorithms showed the lowest MAE in the Asian population (ranging from 6.16 to 6.62) and the highest MAE in the Black population (ranging from 12.17 to 13.84). In the White population, BART, SVR, BRT, MARS and RFR, showed higher mean percentage within 20% and lower MAE than those of MLR (all *p* values <0.05, Table B in S1 File). In the Asian population, no significant difference existed in the MAE and mean percentage within 20% among SVR, BART, BAR, MARS and MLR, these five techniques also performed better than the other algorithms (Table C in S1 File). By contrast, in the Black population, MLR and LAR performed better than any other machine learning based algorithms (all *p* values <0.05; Table D in S1 File). More importantly, the good predictive ability of MARS and BART were stable across the racial groups. MARS and BART showed the lowest MAE and the highest mean percentage within 20% in the White and Asian populations.

### Comparison of Predictive Algorithms within Warfarin Dose Range

Overall, the algorithms provided more accurate prediction in the intermediate- dose range than in the low- or high-dose ranges (Table 3). In the intermediate-dose range, all the algorithms showed mean percentages within 20% in at least 55% of the patients, but a maximum of only 23.79% and 38.94% in the low- and high-dose ranges, respectively.

**Table 3:** Mean absolute error and mean percentage within 20% of actual dose by the therapeutic warfarin dose range in the validation cohort. Data are expressed as mean (95% CI) or percentage.MAE: mean absolute error; MLR: multiple linear regression; SVR: support vector regression; ANN: artificial neural network; RT: regression tree; RFR: random forest regression; BRT: boosted regression tree; MARS: multivariate adaptive regression splines; LAR: lasso regression; BART: Bayesian additive regression trees. The warfarin dose range was divided into three categories based on the 25% and 75% quantiles of WSD in terms of race: in the Asian population: low dose (≤ 14 mg/week), intermediate dose (14–26.25 mg/week), and high dose (> 26.25 mg/week); in the White cohort: low dose (≤22 mg/week), intermediate dose (22–42.49 mg/week), and high dose (>42.49 mg/week); in the Black cohort: low dose (≤30 mg/week), intermediate dose (30–52.5 mg/week), and high dose (>52.5 mg/week); and in the missing or mixed race: low dose (≤22.5 mg/week), intermediate dose (22.5–40 mg/week), and high-dose (>40 mg/week).

Algorithms	Low-dose		Intermediate-dose		High-dose	
	MAE	Within 20%	MAE	Within 20%	MAE	Within 20%
SVR	8.68 (7.82–9.54)	23.79	5.83 (5.39–6.27)	61.55	15.63 (13.63–17.63)	37.29
ANN	9.40 (8.53–10.26)	22.35	6.63 (6.13–7.12)	55.66	16.76 (14.77–18.75)	31.97
RT	9.75 (8.83–10.68)	18.69	6.24 (5.73–6.74)	59.53	16.17 (14.11–18.22)	35.20
MLR	9.60 (8.75–10.45)	17.17	5.63 (5.19–6.08)	63.46	16.36 (14.36–18.37)	32.57
RFR	9.27 (8.42–10.12)	20.02	5.71 (5.27–6.14)	62.85	15.65 (13.63–17.66)	36.44
BRT	9.20 (8.36–10.03)	18.88	5.53 (5.10–5.96)	64.16	15.66 (13.70–17.62)	35.92
MARS	9.03 (8.17–9.89)	21.40	5.59 (5.17–6.01)	63.12	15.27 (13.32–17.22)	38.54
LARS	9.62 (8.78–10.47)	16.97	5.61 (5.17–6.05)	63.65	16.40 (14.39–18.40)	32.33
BART	8.92 (8.06–9.78)	22.48	5.72 (5.28–6.16)	61.72	15.24 (13.28–17.20)	38.94

The performances of certain machine learning-based algorithms were better than that of MLR with regard to warfarin stable dose range (Table 3). LAR performed the same as MLR in the intermediate-dose range (Table 3, Table E in S1 File). In extremely low or high warfarin dose range, six machine learning algorithms, SVR, RT, RFR, BRT, MARS and BART performed better than MLR, with significantly lower MAE and higher mean percentage within 20% (all *p* values <0.05, Tables F and G in S1 File). Compared with MLR, the mean percentage within 20% of these six machine-learning based algorithms increased by 1.52% to 6.62% and 2.63% to 6.37% in the low- and high-dose ranges, respectively (Table 3).

## Discussion

Overall, our study mainly found similar performances of the nine algorithms. However, SVR, MARS and BART provided superior accuracy over MLR in predicting warfarin stable dosage in the whole cohort. In the White population, MARS and BART performed superior. SVR, MARS, MLR, BART and LAR performed statistically indistinguishable and better than any other algorithms in the Asian population, whereas MLR and LAR performed superior in the Black population. In subgroup dose range analysis, six machine learning techniques, SVR, RT, RFR, BRT, MARS and BART performed significantly better than MLR in high- and low- dose ranges.

Performances of the published machine learning-based warfarin pharmacogenetic dosing algorithms were similar in the mixed race and large cohort, compared with that of MLR. SVR, MARS and BART performed better than MLR. However, MLR performed better than ANN, which is inconsistent with the results of previous research. Performances of ANN- and MLR- based on warfarin pharmacogenetic dosing algorithms were compared in previous investigations, and the results showed that ANN performed better than MLR in their cohort [27]. Specifically, the MAEs, after randomly splitting the data as 50% derivation and 50% validation cohort followed by a bootstrap of 200 iterations, were 5.92 and 6.23 mg/week for ANN and MLR respectively. The difference may be ascribed to the following: (i) We used different samples with various characteristics; (ii) We used different software to conduct ANN, although our study was based on R, and C# was used in the previous investigation; (iii) Parameters set for ANN may be different in the two investigations. Compared with the previous investigation, our comprehensive study included five more machine learning algorithms implemented in a larger, racially diverse population, thereby allowing us to draw a general conclusion.

The current preliminary study compared the performances of machine learning techniques with MLR-based warfarin dose algorithms with regard to race. Interestingly, in the White population, some machine learning techniques performed better than MLR; in the Asian population, BART, SVR, MARS, LAR and MLR performed similarly. By contrast, in the Black population, MLR and LAR showed optimal performance. These findings may be attributed to the difference in genetic and non-genetic characteristics of the racial groups, not to mention differences in sample size. The size of White, Asian and Black populations were 2718, 1156 and 665, respectively. Considering that machine learning techniques concern the construction and study of systems that can be learned from training data, a general model about this space will produce sufficiently accurate predictions in new cases [18]. Thus, more information supplied by the training data will improve accuracy. In addition, machine learning techniques are designed for large data; thus, these methods rely greatly on sample sizes compared with MLR [17, 18].

Our results indicated that the mean percentages within 20% of all the studied algorithms do not differ in terms of race, whereas the average MAEs do. The greatest difference in the average MAE was 7.29 mg/week, which was observed between the Black and Asian populations. The greatest difference in the mean percentage within 20% was also observed between these two populations at about 4.97% only. These results may suggest that the Black cohorts demonstrated the highest variability in warfarin dose requirements among three racial groups. The mean (standard deviation) of warfarin stable dosage in Blacks was 42.85 (18.71) mg/week, versus 34.39 (17.58) mg/week in Whites and 21.49 (10.00) mg/week in Asians.

Subgroup analysis on warfarin stable dose range reflected the advantages of machine learning techniques in extreme dosage range predictions, although the warfarin dose category for a specific patient was unknown before clinical practice. Our findings indicated that the nine algorithms exhibited a lower MAE and a higher mean percentage within 20% in the intermediate-dose range than those in the high- and low- dose ranges. However, notably, the intermediate-dose group was least likely to benefit from pharmacogenetics. Therefore, better prediction did not present real clinical benefit to the group. In the low- and high-dose ranges, six of the eight machine learning techniques (SVR, RT, RFR, BRT, MARS and BART) performed better than MLR. These findings may be ascribed to the capacity of machine learning techniques to assess the characteristics of patients under extreme dosage range. However, MLR is designed to assess patients on an intermediate warfarin dose, which is the case of most patients included in this study.

The explanation behind the relatively unusual but efficient overall performance of the machine learning techniques in extremely low and high dosage subgroups should be explored. Notably, the underlying relationship between the dependent variable (optimal stable dose of warfarin) and independent variables (genetic and non-genetic covariates) is complex, and gene–gene and gene–environment interactions may exist [21, 30]; moreover, no reliable *a priori* statistical model is available. Machine learning techniques can deal with inferential problems, such as collinear interactions among variables, outliers, and hidden variables owing to their ability to self-adjust their structure as they encounter errors, irrespective of their underlying degree nonlinearity, machine learning can handle numerous variables simultaneously, leading to structurally robust results, regardless if the background of statistical process is not well understood [31–33].

Few limitations are noted in our study. First, this retrospective study used the pre-existing IWPC database. The cohorts comprised a mixed population which coming from different countries, regions and clinical research sites, which may have led to classification bias by introducing a huge variability in genotypes. Second, given that the sample sizes of Black and Asian are much smaller than that of White, a potential effect may arise in the comparison based on race. Thus, our results should be validated and replicated in future research with a larger sample size. Third, we were not able to evaluate the performances of all prediction algorithms exclusively. Alternatively, we conducted our research by using the methods in the publications included in this study. Therefore, more comprehensive studies on the evaluation of the nine techniques presented, along with many other techniques, should be conducted in the near future before general conclusions can be drawn about the superiority of a particular approach.

## Conclusion

In this systematic comparison, the published machine learning and MLR based warfarin pharmacogenetic algorithms generally performed similarly. Some machine learning-based algorithms performed significantly better than MLR in the White population, but not in the Asian and Black populations; Machine learning techniques also performed better in the low- and high-dose ranges, but not in the intermediate-dose range, as indicated by the low MAE and high percentage within 20% values.

## Supporting Information

S1 File Statistical significance between ideal rate and MAE of all algorithms obtained with *t*-tests in the whole validation cohort with regard to race and warfarin dose group. (Tables A to G in S1 File).
(PDF)
