# Supplementary material for: Comparison of Nine Statistical Model Based Warfarin Pharmacogenetic Dosing Algorithms Using the Racially Diverse International Warfarin Pharmacogenetic Consortium Cohort Database
Source: PLoS One. 2015 Aug 25;10(8):e0135784. doi: 10.1371/journal.pone.0135784 (PMC4549222; doi:10.1371/journal.pone.0135784)
Supplement: S1 File — (Tables A to G in S1 File). (PDF) [file pone.0135784.s001.pdf]

Table A Statistical significance between ideal rate of all algorithms in validation cohort (n=960)

Algorithms	ANN	RT	MLR	RFR	BRT	MARS	LAR	BART
**SVR**	3.52×10^{-23}	1.93×10^{-29}	4.87×10^{-19}	1.09×10^{-2}	1.45×10^{-1}	1.70×10^{-2}	7.82×10^{-19}	4.81×10^{-1}
**ANN**		8.99×10^{-6}	4.98×10^{-11}	1.46×10^{-19}	4.93×10^{-21}	2.68×10^{-26}	6.76×10^{-11}	5.04×10^{-24}
**RT**			1.06×10^{-5}	6.11×10^{-21}	8.41×10^{-24}	3.71×10^{-37}	1.84×10^{-5}	8.00×10^{-30}
**MLR**				7.13×10^{-11}	1.37×10^{-13}	2.14×10^{-27}	9.38×10^{-1}	1.26×10^{-19}
**RFR**					2.93×10^{-1}	1.54×10^{-6}	8.10×10^{-11}	1.93×10^{-3}
**BRT**						1.90×10^{-4}	1.68×10^{-13}	3.87×10^{-2}
**MARS**							5.10×10^{-27}	1.19×10^{-1}
**LAR**								1.84×10^{-19}

Data are presented as the p-values of two independent samples t-test between the predicted ideal rates of all algorithms.

MLR: multiple linear regression; SVR: support vector regression; ANN: artificial neural network; RT: regression tree; RFR: random forest regression; BRT: boosted regression tree; MARS: multivariate adaptive regression splines; LAR: lasoo regression; BART: Bayesian additive regression trees.

Table B Statistical significance between ideal rate of all algorithms in the White validation cohort (n=544)

Algorithms	ANN	RT	MLR	RFR	BRT	MARS	LAR	BART
**SVR**	1.65×10^{-18}	1.07×10^{-36}	6.68×10^{-14}	1.69×10^{-4}	1.41×10^{-11}	3.27×10^{-6}	1.07×10^{-14}	9.75×10^{-5}
**ANN**		3.66×10^{-1}	3.96×10^{-7}	3.35×10^{-13}	2.25×10^{-16}	2.85×10^{-25}	1.16×10^{-6}	3.11×10^{-24}
**RT**			1.04×10^{-11}	1.29×10^{-25}	4.78×10^{-32}	2.20×10^{-50}	1.20×10^{-10}	9.33×10^{-47}
**MLR**				1.35×10^{-5}	3.41×10^{-10}	3.63×10^{-27}	7.20×10^{-1}	4.30×10^{-24}
**RFR**					2.13×10^{-2}	1.66×10^{-15}	3.17×10^{-6}	6.77×10^{-13}
**BRT**						2.69×10^{-9}	6.03×10^{-11}	2.16×10^{-7}
**MARS**							6.60×10^{-28}	5.34×10^{-1}
**LAR**								7.25×10^{-25}

Data are presented as the p-values of two independent samples t-test between the predicted ideal rates of all algorithms.

MLR: multiple linear regression; SVR: support vector regression; ANN: artificial neural network; RT: regression tree; RFR: random forest regression; BRT: boosted regression tree; MARS: multivariate adaptive regression splines; LAR: lasoo regression; BART: Bayesian additive regression trees.

Table C Statistical significance between ideal rate of all algorithms in the Asian validation cohort (n=231)

Algorithms	ANN	RT	MLR	RFR	BRT	MARS	LAR	BART
**SVR**	1.21×10^{-9}	2.34×10^{-16}	5.29×10^{-1}	3.23×10^{-3}	6.89×10^{-3}	7.57×10^{-1}	4.30×10^{-1}	4.56×10^{-1}
**ANN**		3.91×10^{-1}	1.46×10^{-8}	6.25×10^{-5}	1.88×10^{-5}	3.60×10^{-10}	3.08×10^{-8}	3.61×10^{-11}
**RT**			1.03×10^{-14}	6.15×10^{-9}	6.47×10^{-10}	4.11×10^{-17}	3.66×10^{-14}	6.77×10^{-19}
**MLR**				1.94×10^{-2}	3.78×10^{-2}	3.49×10^{-1}	8.70×10^{-1}	1.64×10^{-1}
**RFR**					7.51×10^{-1}	1.23×10^{-3}	3.06×10^{-2}	1.92×10^{-4}
**BRT**						2.72×10^{-3}	5.77×10^{-2}	4.55×10^{-4}
**MARS**							2.75×10^{-1}	6.71×10^{-1}
**LAR**								1.22×10^{-1}

Data are presented as the p-values of two independent samples t-test between the predicted ideal rates of all algorithms.

MLR: multiple linear regression; SVR: support vector regression; ANN: artificial neural network; RT: regression tree; RFR: random forest regression; BRT: boosted regression tree; MARS: multivariate adaptive regression splines; LAR: lasoo regression; BART: Bayesian additive regression trees.

Table D Statistical significance between ideal rate of all algorithms in the Black validation cohort (n=133)

Algorithms	ANN	RT	MLR	RFR	BRT	MARS	LAR	BART
**SVR**	4.15×10^{-4}	7.45×10^{-16}	3.76×10^{-2}	2.18×10^{-1}	9.33×10^{-1}	9.15×10^{-1}	2.73×10^{-2}	3.79×10^{-1}
**ANN**		2.84×10^{-5}	1.96×10^{-7}	1.56×10^{-2}	9.83×10^{-4}	4.96×10^{-4}	1.10×10^{-7}	5.31×10^{-3}
**RT**			1.36×10^{-21}	3.82×10^{-12}	2.59×10^{-14}	6.78×10^{-16}	5.01×10^{-22}	1.67×10^{-13}
**MLR**				1.26×10^{-3}	4.27×10^{-2}	2.65×10^{-2}	9.02×10^{-1}	3.38×10^{-3}
**RFR**					2.82×10^{-1}	2.51×10^{-1}	8.10×10^{-4}	7.07×10^{-1}
**BRT**						9.89×10^{-1}	3.19×10^{-2}	4.60×10^{-1}
**MARS**							1.88×10^{-2}	4.31×10^{-1}
**LAR**								2.22×10^{-3}

Data are presented as the p-values of two independent samples t-test between the predicted ideal rates of all algorithms.

MLR: multiple linear regression; SVR: support vector regression; ANN: artificial neural network; RT: regression tree; RFR: random forest regression; BRT: boosted regression tree; MARS: multivariate adaptive regression splines; LAR: lasoo regression; BART: Bayesian additive regression trees.

Table E Statistical significance between ideal rate of all algorithms in validation cohort (Intermediate-dose group)

Algorithms	ANN	RT	MLR	RFR	BRT	MARS	LAR	BART
**SVR**	8.66×10^{-11}	6.58×10^{-9}	6.01×10^{-10}	6.49×10^{-5}	3.02×10^{-14}	2.86×10^{-7}	1.76×10^{-11}	6.24×10^{-1}
**ANN**		8.49×10^{-6}	3.95×10^{-16}	2.56×10^{-14}	5.01×10^{-18}	3.57×10^{-15}	1.15×10^{-16}	3.72×10^{-11}
**RT**			1.25×10^{-26}	6.19×10^{-19}	1.34×10^{-30}	3.13×10^{-23}	2.42×10^{-28}	1.73×10^{-9}
**MLR**				4.04×10^{-2}	1.84×10^{-2}	2.14×10^{-1}	4.75×10^{-1}	4.74×10^{-8}
**RFR**					6.26×10^{-5}	3.63×10^{-1}	7.46×10^{-2}	7.27×10^{-4}
**BRT**						6.00×10^{-4}	8.69×10^{-2}	4.07×10^{-12}
**MARS**							5.27×10^{-2}	9.35×10^{-6}
**LAR**								2.09×10^{-9}

Data are presented as the p-values of two independent samples t-test between the predicted ideal rates of all algorithms.

MLR: multiple linear regression; SVR: support vector regression; ANN: artificial neural network; RT: regression tree; RFR: random forest regression; BRT: boosted regression tree; MARS: multivariate adaptive regression splines; LAR: lasoo regression; BART: Bayesian additive regression trees.

Table F Statistical significance between ideal rate of all algorithms in validation cohort (low-dose group)

Algorithms	ANN	RT	MLR	RFR	BRT	MARS	LAR	BART
**SVR**	1.84×10^{-1}	7.86×10^{-27}	1.50×10^{-51}	2.94×10^{-24}	1.06×10^{-30}	1.95×10^{-10}	2.16×10^{-53}	2.18×10^{-4}
**ANN**		1.16×10^{-3}	4.42×10^{-6}	3.21×10^{-2}	1.76×10^{-3}	3.80×10^{-1}	2.02×10^{-6}	9.06×10^{-1}
**RT**			1.28×10^{-4}	8.95×10^{-4}	6.58×10^{-1}	1.00×10^{-9}	1.62×10^{-5}	9.49×10^{-17}
**MLR**				3.40×10^{-17}	1.28×10^{-6}	4.49×10^{-26}	5.09×10^{-1}	6.98×10^{-37}
**RFR**					1.17×10^{-3}	1.03×10^{-4}	4.17×10^{-19}	9.11×10^{-12}
**BRT**						2.48×10^{-10}	7.58×10^{-8}	1.51×10^{-18}
**MARS**							7.26×10^{-28}	3.87×10^{-3}
**LAR**								1.01×10^{-38}

Data are presented as the p-values of two independent samples t-test between the predicted ideal rates of all algorithms.

MLR: multiple linear regression; SVR: support vector regression; ANN: artificial neural network; RT: regression tree; RFR: random forest regression; BRT: boosted regression tree; MARS: multivariate adaptive regression splines; LAR: lasoo regression; BART: Bayesian additive regression trees.

Table G Statistical significance between ideal rate of all algorithms in validation cohort (high-dose group)

Algorithms	ANN	RT	MLR	RFR	BRT	MARS	LAR	BART
**SVR**	4.51×10^{-4}	2.48×10^{-6}	4.20×10^{-26}	4.42×10^{-2}	1.32×10^{-3}	2.36×10^{-3}	1.67×10^{-27}	6.08×10^{-5}
**ANN**		3.10×10^{-2}	6.85×10^{-1}	3.07×10^{-3}	8.47×10^{-2}	1.94×10^{-5}	8.10×10^{-1}	6.58×10^{-6}
**RT**			1.13×10^{-9}	6.13×10^{-2}	1.05×10^{-1}	4.52×10^{-13}	6.89×10^{-11}	8.85×10^{-16}
**MLR**				3.60×10^{-18}	9.11×10^{-15}	4.25×10^{-36}	5.11×10^{-1}	5.48×10^{-40}
**RFR**					2.41×10^{-1}	1.32×10^{-6}	1.49×10^{-19}	9.88×10^{-9}
**BRT**						2.60×10^{-9}	3.99×10^{-16}	8.74×10^{-12}
**MARS**							2.20×10^{-37}	3.30×10^{-1}
**LAR**								3.30×10^{-41}

Data are presented as the p-values of two independent samples t-test between the predicted ideal rates of all algorithms.

MLR: multiple linear regression; SVR: support vector regression; ANN: artificial neural network; RT: regression tree; RFR: random forest regression; BRT: boosted regression tree; MARS: multivariate adaptive regression splines; LAR: lasoo regression; BART: Bayesian additive regression trees.
